# Predictors of student mask mandate policies in United States school districts during the COVID-19 pandemic

**DOI:** 10.3389/fpubh.2023.1217638

**Published:** 2023-07-31

**Authors:** Lauren M. Klein, Sara B. Johnson, Annette C. Anderson, Kelly Beharry, Ruth Faden, Xinxing Guo, Medha Kallem, Andrew Nicklin, Alan Regenberg, Azka Tariq, Megan E. Collins

**Affiliations:** ^1^Department of Pediatrics, Johns Hopkins University School of Medicine, Baltimore, MD, United States; ^2^Department of Population, Family, and Reproductive Health, Johns Hopkins University Bloomberg School of Public Health, Baltimore, MD, United States; ^3^Department of Mental Health, Johns Hopkins University Bloomberg School of Public Health, Baltimore, MD, United States; ^4^Johns Hopkins University School of Education, Baltimore, MD, United States; ^5^University of Michigan Medical School, Ann Arbor, MI, United States; ^6^Wilmer Eye Institute, Johns Hopkins University School of Medicine, Baltimore, MD, United States; ^7^Johns Hopkins Berman Institute of Bioethics, Baltimore, MD, United States; ^8^Department of Health Policy and Management, Johns Hopkins University Bloomberg School of Public Health, Baltimore, MD, United States; ^9^Bloomberg Center for Government Excellence, Baltimore, MD, United States; ^10^Department of Ophthalmology, Johns Hopkins University School of Medicine, Baltimore, MD, United States

**Keywords:** school health, COVID-19, mask policy, public health, health promotion

## Abstract

**Introduction:**

Although factors such as urbanicity, population demographics, and political affiliation have been linked with COVID-19 masking behavior and policy in community settings, little work has investigated factors associated with school mask policies. We sought to characterize United States state and school district student COVID-19 masking policies during the 2021–22 school year and determine predictors of these mandates at four time points, including before and after federal guidance relaxed school mask recommendations in February 2022.

**Methods:**

Student mask policies for US states and the District of Columbia, as well as a sample of 56 districts were categorized as prohibited, recommended, or required in September 2021, November 2021, January 2022, and March 2022 based on the Johns Hopkins eSchool+ Initiative School Reopening Tracker. Changes in policies over time were characterized. Generalized estimating equations and logistic regression were used to evaluate whether political affiliation of governor, urbanicity, economic disadvantage, and race/ethnic composition of district students, and county-level COVID-19 incidence predicted the presence of a district mask mandate at any time point and at all four time points.

**Results:**

State and district policies changed over time. Districts that implemented student mandates at any point were more likely to be in states with Democratic governors (AOR: 5.52; 95% CI: 2.23, 13.64) or in non-rural areas (AOR: 8.20; 95% CI: 2.63, 25.51). Districts that retained mask mandates at all four time points were more likely to have Democratic governors (AOR: 5.39; 95% CI: 2.69, 10.82) and serve a smaller proportion of economically disadvantaged students (AOR: 0.97; 95% CI: 0.95, 0.99). Districts serving a larger proportion of students from minoritized racial/ethnic groups were more likely to have mask mandates at any or all timepoints. Notably, county-level COVID-19 prevalence was not related to the presence of a mask mandate at any or all time points. By March 2022, no factors were significantly associated with district mask policy.

**Discussion:**

Political, geographic, and demographic characteristics predicted the likelihood of student mask mandates in the 2021–22 school year. Public health promotion messages and policy must account for variation in these factors, potentially through centralized and consistent messaging and unbiased, trustworthy communication.

## 1. Introduction

In response to on-going concerns about COVID-19, in July 2021, the Centers for Disease Control and Prevention (CDC) recommended universal masking in kindergarten through grade 12 schools during most of the 2021–2022 school year for students, teachers and visitors, regardless of individuals’ vaccination status or community transmission rates (1, 2). At some point during the 2021–2022 school year, 18 states and the District of Columbia implemented mask mandates for public (and in some cases, private) schools in their jurisdiction (3–5), while other states did not enact mask mandates or expressly prohibited them.

In February 2022, however, the CDC updated its guidance to recommend masking in schools only in the context of high community transmission or strained healthcare system capacity (3, 6).

Following this change in CDC masking guidance, many of the 19 states (including the District of Columbia) rescinded previously implemented mask mandates in schools (3). In response to state-level policy changes, some school districts lifted mandates while others chose to retain them. A recent study in Massachusetts, the only study so far investigating factors associated with school district decisions on mask mandates, found that school districts that lifted mask mandates experienced greater incidence of COVID-19 among students and staff compared to those that did not (4). Moreover, districts that continued their mask mandates after the statewide mandate was lifted were more likely to serve students from minoritized racial and ethnic groups and students in poverty (4). To date, however, factors associated with mandates in districts across the US have not been evaluated.

The impact of COVID-19 has varied substantially across communities in the United States. In light of findings from the Massachusetts study, it is possible that, nationwide, communities that bore a greater burden or COVID-19 related morbidity and mortality risk adopted were more likely to adopt more stringent masking policies; communities of color and lower socioeconomic status have been disproportionately impacted (7, 8). Urban and rural areas have also been impacted differently; while cases overall were initially concentrated in urban areas, intensity shifted between urban and rural areas over time (9).

Prior research at the national level suggests that individual characteristics like political affiliation (10), race (10, 11), gender (11), urbanicity (10), and income (10) are associated with the acceptability of masking as a public health strategy and adherence to masking requirements. Limited research has also identified factors associated with the presence of statewide mask mandates, notably political party affiliation of the state’s governor; states with Republican governors have been less likely to implement statewide mask mandates (12, 13).

The objective of this study was to characterize student mask policies for all 50 US states and the District of Columbia (DC) and a sample of United States school districts at four points during the 2021–2022 school year: September 2021, November 2021, January 2022, and March 2022. We then sought to determine whether urbanicity, demographics of district students, political affiliation of the state governor, or community viral transmission levels predicted the presence of a school mask mandate at the school district level at any time point. Finally, we examined whether the factors that predicted a mask mandate at any time point differed from those that predicted retaining a mask mandate at all four time points, including after the CDC’s masking guidance was revised. We hypothesized that school districts in states with Democratic governors, those in urban areas, those with a higher proportion of students from minoritized racial/ethnic groups or economically disadvantaged students, and higher county-level COVID-19 transmission levels would be more likely to have mask mandates. We also hypothesized that district demographic composition would be more strongly associated with the likelihood of the most conservative masking approach (i.e., requiring student masking at all four time points) as compared to the likelihood of having a mask mandate at any time point.

## 2. Materials and methods

### 2.1. State sample

Publicly available reopening plans published after July 26, 2021 on state department of education websites were coded and analyzed (14). Data from all 50 states and the District of Columbia were extracted from the Johns Hopkins eSchool+ Initiative 2021–22 School Reopening Tracker (SRT) (14). In this analysis, District of Columbia was regarded as a state and a total of 51 states were included. This study was determined to be non-human subjects research, and was thus approved as exempt research by the Johns Hopkins University Institutional IRB.

### 2.2. District sample

School districts were sampled from the Johns Hopkins eSchool+ Initiative 2021–22 SRT. Sampling has been described in detail elsewhere (14). Briefly, to capture geographic diversity, two states were randomly selected from the eight US geographic regions defined by the Bureau of Economic Analysis, except in the cases of the Rocky Mountain Region (one state) because of inclusion criteria and the Southeast region (three states) because of the number of states in that region (14). The four most populous states based on 2021 data (15) (New York, Florida, Texas, and California) were also included (14). Within each state, eligible districts were identified for inclusion in the dataset; a school district needed to have a publicly available reopening plan that described its masking policy by September 2, 2021 and serve at least 500 students ages 5–17 to be considered (14). Of this pool of eligible districts, within each of the identified states, the largest, maximum poverty, and minimum poverty school districts were chosen (14). Maximum and minimum poverty was characterized from Small Area Income and Poverty Estimates (SAIPE) data (16), and the largest district was identified from National Center for Education Statistics data (17). Exceptions were Hawaii and the District of Columbia, both of which have only one school district for their entire jurisdiction (14). In total, 56 United States school districts were included.

### 2.3. Dependent variable: state and district mask mandates

The dependent variable was the presence of a student mask mandate assessed at four time points: September 2021 (beginning of 2021–22 school year), November 2021, January 2022, and March 2022 (immediately after CDC masking recommendation for schools were updated). At the state level, school mask mandates were classified as “Prohibited” (i.e., the state had expressly banned mask mandates in schools), “Required” (i.e., the state had a mandate for one or more student groups), or “Recommended/Optional” (i.e., the state recommended but did not require masks for any student group). At the school district level, mandates were similarly classified as “Prohibited,” “Required,” or “Recommended/Optional” based on published school district policies. For cases in which a policy was under litigation, it was classified based on the most recent publicly available information from the state or district level department of education website. The current analysis focuses on mask policies for students. In preliminary analyses, we evaluated policies for teachers and school staff, and found that policies and results were consistent for teachers and students. However, results for teacher mask mandates from the same state and district samples are provided in Supplementary Tables 2, 3.

### 2.4. Independent variables

#### 2.4.1. Political affiliation

Political affiliation of the governor (or mayor, in the case of the District of Columbia) was categorized as Democrat, Republican, or Independent based on data from the National Governors Association (18). All governors in the study sample were affiliated with Democrat or Republican parties.

#### 2.4.2. District urbanicity

School district urbanicity was extracted from the 2020 to 2021 National Center for Education Statistics Elementary/Secondary Information System (17). Districts were coded as rural, urban, suburban, or town and then collapsed into a rural/urban binary variable based on the guidance by the National Center for Education Statistics (19).

#### 2.4.3. District race/ethnic composition

Racial/ethnic composition was calculated as the proportion of students in the district from racial/ethnic groups other than white Non-Hispanic from the National Center for Education Statistics (17). The continuous variable was broken into quartiles for inclusion in statistical models.

#### 2.4.4. Students with economic disadvantage

The percent of economically disadvantaged students in each district in 2020 was from the Stanford Education Data Archive (SEDA) (20). SEDA sourced this data from EdFacts (21), which defines economic disadvantage as the number of students who meet the state criteria for classification as economically disadvantaged according to the state definition (22).

#### 2.4.5. Relative county-level COVID-19 burden

County-level 14-day average new COVID-19 cases (per 1,000) were from the Johns Hopkins Coronavirus Resource Center (23). Cases were categorized into tertiles (high, medium, and low), based on values for each month.

### 2.5. Statistical analysis

First, we described the distribution of mask policies for all 51 states and for the 56 school districts at each of the four time points and changes over time. Then, we used generalized estimating equations with exchangeable correlation structure to determine whether urbanicity, demographics of district students, political affiliation of the state governor, or community viral transmission levels predicted the presence of a school mask mandate at any time point, taking into account the correlation among the districts over time.

Next, we examined whether the same district-level factors predicted having a mask mandate at all four time points using logistic regression models. Finally, in sensitivity analyses, we used separate multivariable logistic regression models at each time point to explore whether the cross-sectional relationship between district-level factors and the presence of a mask mandate changed over time, particularly after the CDC recommendations for masking in schools changed.

This study was determined to be exempt by The Johns Hopkins School of Medicine Institutional Review Board.

## 3. Results

### 3.1. State mask policies

Overall, 19 states (37%) had a student mask mandate at any point; 15 of these (79%) lifted the mandate at some point during the school year, and 4 (21%) states required masks at all four time points. 27 states (53%) never implemented a mandate. Five states (10%) prohibited a mask mandate at any point, the majority of which (80%; *n* = 4) shifted from prohibiting a mask mandate to recommending one.

**Figure 1:**
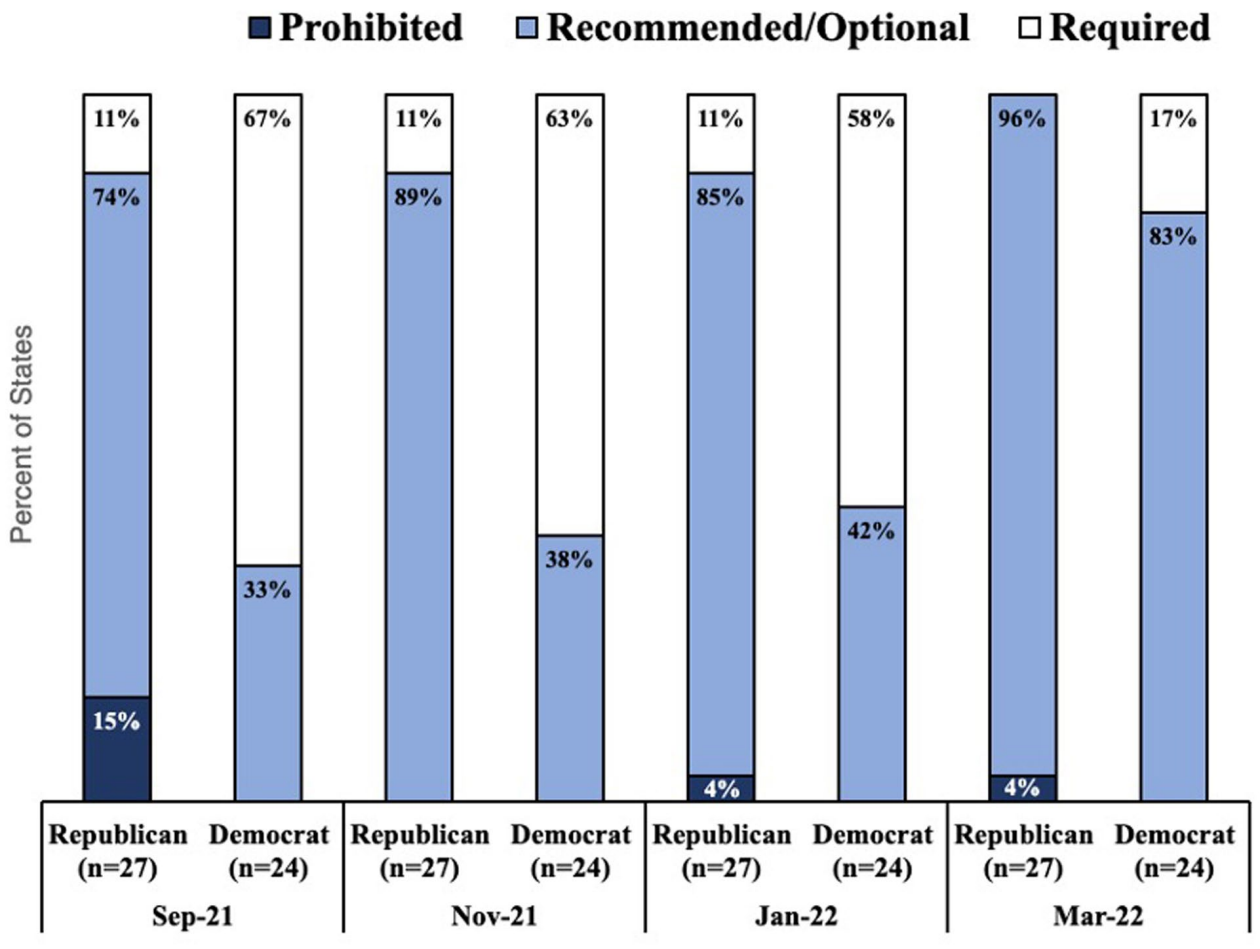
Proportion of states with democratic and republican governors who prohibited, recommended, or required masks in September 2021, November 2021, January 2022, and March 2022 by governor’s political affiliation.

As shown in Figure 1, states with Democratic governors were more likely to have a mask mandate, and states with Republican governors were more likely to either recommend or make optional mask wearing, or expressly prohibit mask mandates. The proportion of Republican states requiring a mandate was relatively consistent whereas the proportion of Democratic states with mask mandates decreased over time. Notably, between January and March 2022, the proportion of states with Democratic governors that had a mask mandate decreased from 58 to 17% (Figure 1).

### 3.2. District mask mandates

**Figure 2:**
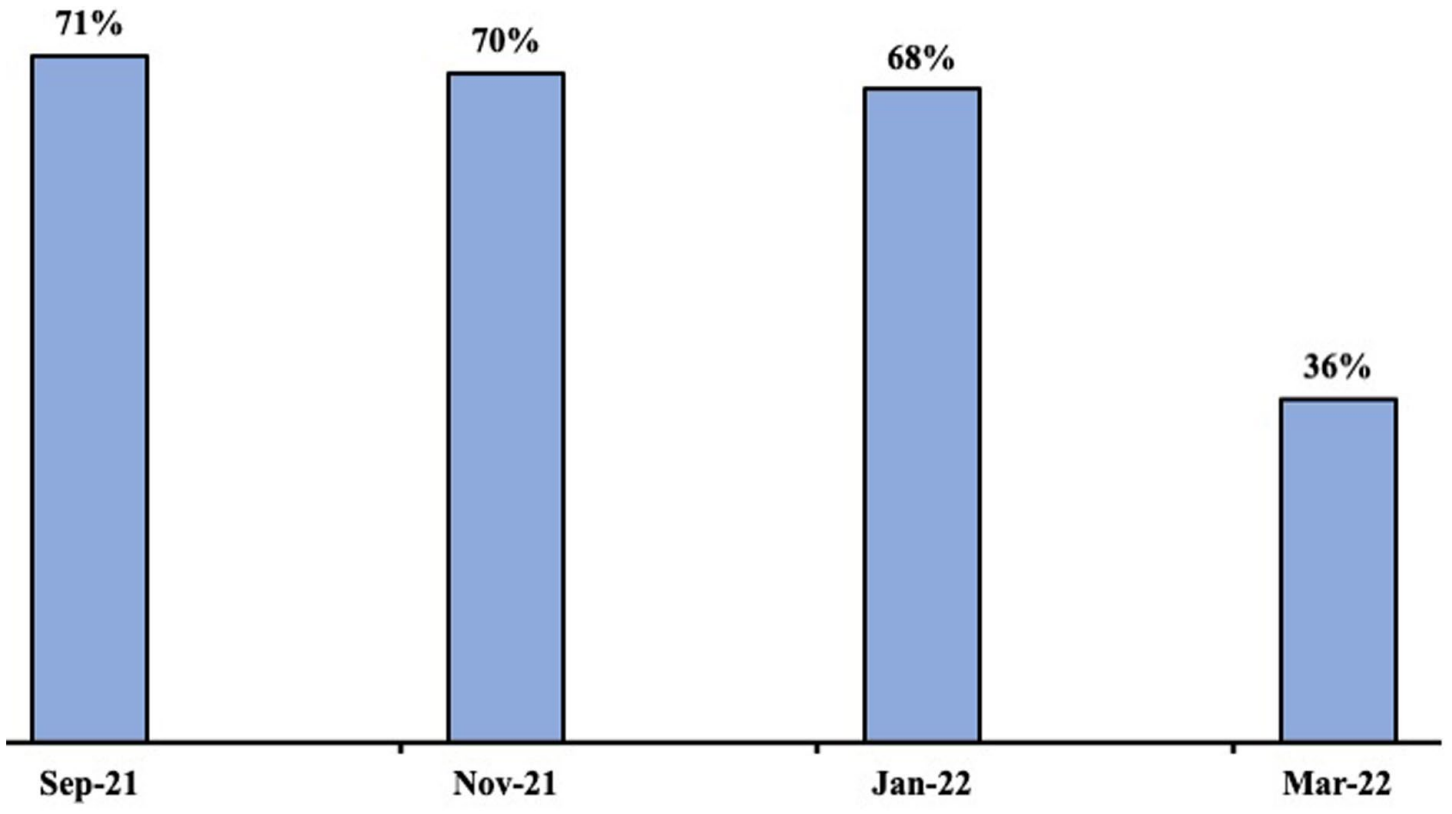
Proportion of school districts with student mask mandates in September 2021, November 2021, January 2022, and March 2022.

The proportion of school districts with mask mandates remained relatively consistent from September 2021 through January 2022 (Figure 2). However, between January 2022 and March 2022, there was a 47% relative decrease in the prevalence, from 68 to 36%, of student mask mandates.

A summary of the characteristics of the 56 United States school districts included in the sample is shown in Table 1. The majority of districts were in non-rural areas (79%); on average, districts served student populations where 58% of students belonged to a racial/ethnic minority and just under two thirds (64.8%) of students were economically disadvantaged. Of the 56 districts, 17 (30%) had a mask mandate at all four time points, 27 (48%) made changes to their mask mandate policies during the study period (89% of which were to rescind a mandate), and 12 (21%) never implemented a mask mandate.

**Table 1:** School district characteristics by student mask policy categories over four time points of September 2021, November 2021, January 2022, and March 2022.

	Total	Required	Recommended ^c^ → Required	Required → Recommended ^a^^,^^c^	Recommended ^c^
*N*	56	17	3	24	12
Governor affiliation, *n* (%)^b^					
*Democrat*	26 (46)	12 (71)	0 (0)	13 (54)	1 (8)
*Republican*	30 (54)	5 (29)	3 (100)	11 (46)	11 (92)
Urbanicity, *n* (%)^b^					
*Rural*	12 (21)	2 (12)	0 (0)	2 (8)	8 (67)
*Non-rural*	44 (79)	15 (88)	3 (100)	22 (92)	4 (33)
Students from minoritized race/ethnic group %, median (25, 75th percentile)	58.5 (26.2, 86.0)	83.4 (44.8, 88.7)	19.0 (15.6, 99.9)	74.8 (36.4, 86.6)	34.4 (17.0, 48.4)
Students with economic disadvantage %, median (25, 75th percentile)	64.8 (21.4, 82.0)	71.1 (30.2, 85.6)	17.6 (9.2, 95.3)	65.5 (37.4, 81.7)	46.9 (21.4, 73.5)
Relative county-level COVID-19 burden in September 2021, *n* (%)^b^					
*Low*	19 (34)	9 (53)	2 (67)	6 (25)	2 (17)
*Medium*	19 (34)	5 (29)	1 (33)	11 (46)	2 (17)
*High*	18 (32)	3 (18)	0 (0)	7 (29)	8 (66)

a One district (Omaha Public Schools district, NE) changed twice (recommended → required → recommended). They were assigned to the required → recommended category.

b Statistically significant differences between mask policy groups at *p* < 0.05 level.

c Recommended or optional masking.

### 3.3. District factors predicting the presence of a mask mandate at any time point

First, we assessed the independent relationships between school district characteristics of interest and the presence of a school district mask mandate at any of the four time points. Having a Democratic governor (OR: 4.83; 95% CI: 2.08, 11.24) and being in a non-rural area (OR: 7.35; 95% CI: 2.04, 26.51) were independently associated with greater odds of a mandate at any time point (Table 2). In multivariable models, districts in states with a Democratic governor were 5.52 times (95% CI: 2.23, 13.64) as likely as those in states with a Republican governor, and districts in urban areas were 8.20 times (95% CI: 2.63, 25.51) as likely as those in rural areas, to have implemented a student mask mandate at any time point (Table 2). Having an increasing proportion of students from minoritized racial/ethnic groups was qualitatively associated with greater odds of a mandate; however, this was only statistically significant in the third quartile in the multivariable models (AOR: 4.63; 95% CI: 1.21, 17.78; Table 2).

**Table 2:** Univariate and multivariable associations between district level characteristics and the odds of having a school district level mask mandate at any one time point (September 2021, November 2021, January 2022, or March 2022).

	Student mask mandate			
	Univariate		Multivariable ^b^	
	OR	95% CI	AOR	95% CI
Governor affiliation^a^				
*Republican*	Reference	--	Reference	--
*Democrat*	**4.83**	**2.08, 11.24**	**5.52**	**2.23, 13.64**
Urbanicity^a^				
*Rural*	Reference	--	Reference	--
*Non-rural*	**7.35**	**2.04, 26.51**	**8.20**	**2.63, 25.51**
Students from minoritized race/ethnic group %^a^				
*First quartile (7.1– 26.0%)*	Reference	--	Reference	--
*Second quartile (26.5–55.6%)*	1.00	0.31, 3.26	0.60	0.18, 2.03
*Third quartile (61.3–85.2%)*	3.22	0.97, 10.74	**4.63**	**1.21, 17.78**
*Fourth quartile (86.8–99.9%)*	2.94	0.87, 9.92	5.37	0.96, 29.95
Students with economic disadvantage %	1.00	0.99, 1.02	0.99	0.97, 1.01
Relative county-level COVID-19 burden				
*Low*	Reference	--	Reference	--
*Medium*	1.39	0.75, 2.56	1.54	0.63, 3.73
*High*	1.64	0.99, 2.72	2.00	0.97, 4.14

OR, odds ratio; CI, confidence interval; AOR, adjusted odds ratio; QIC, quasilikelihood under the independence model criterion.

a Bold font indicates statistically significant associations at *p* < 0.05.

b Multivariable generalized estimating equation model QIC = 251.125.

### 3.4. District factors predicting the presence of a mask mandate at all time points

In univariate logistic regression models assessing factors predicting the presence of a district student mask mandate at all four of the time points (Table 3), the role of state governor affiliation, urbanicity, and race/ethnic composition of the district were similar to models predicting the presence of a mandate at any time point (Table 2). In multivariable models, presence of a Democratic governor was associated with 5.39 times greater odds of a mask mandate (95% CI: 2.69, 10.82). In addition, being in the third (AOR: 9.58; 95% CI: 1.85, 49.56) or fourth (AOR: 16.15; 95% CI: 2.35, 110.74) highest quartiles of the district racial/ethnic composition variable was significantly associated with increased odds of a mask mandate (Table 3), and having a greater proportion of economically disadvantaged students was associated with lower odds of a student mask mandate (AOR: 0.97; 95% CI: 0.95, 0.99; Table 3). Urbanicity was not significantly associated with having a mandate at all four time points in multivariable models.

**Table 3:** Univariate associations and fully-adjusted multivariable associations between district level characteristics and the odds of having a school district level mask mandate policy at all four time points (September 2021, November 2021, January 2022, and March 2022).

	Student mask mandate			
	Univariate		Multivariable ^b^	
	OR	95% CI	AOR	95% CI
Governor affiliation ^a^				
*Republican*	Reference	--	Reference	--
*Democrat*	**4.29**	**2.32, 7.93**	**5.39**	**2.69, 10.82**
Urbanicity^a^				
*Rural*	Reference	--	Reference	--
*Non-rural*	**2.59**	**1.14, 5.88**	1.64	0.61, 4.42
Students from minoritized race/ethnic group %^a^				
*First quartile (7.1–26.0%)*	Reference	--	Reference	--
*Second quartile (26.5–55.6%)*	0.61	0.23, 1.63	0.57	0.18, 1.80
*Third quartile (61.3–85.2%)*	**2.75**	**1.20, 6.30**	**9.58**	**1.85, 49.56**
*Fourth quartile (86.8–99.9%)*	**2.75**	**1.20, 6.30**	**16.15**	**2.35, 110.74**
Students with economic disadvantage %	1.01	1.00, 1.02	**0.97**	**0.95, 0.99**
Relative county-level COVID-19 burden				
*Low*	Reference	--	Reference	--
*Medium*	0.59	0.30, 1.20	0.65	0.29, 1.46
*High*	0.81	0.41, 1.62	0.88	0.40, 1.94

OR, odds ratio; CI, confidence interval; AOR, adjusted odds ratio.

a Bold font indicates statistically significant associations at *p* < 0.05 level.

b Multivariable regression model pseudo-*R*^2^ = 0.1445.

### 3.5. Sensitivity analysis

To explore potential differences in the relationship between district characteristics and the presence of a mask mandate at each time point, in sensitivity analyses, we used multivariable logistic regression models. Governor political affiliation was consistently associated with the presence of a mask mandate until March (Supplementary Table 1). Notably, in March, none of the factors assessed were significantly associated with the presence of a district mask policy (Supplementary Table 1).

## 4. Discussion

In this study, we aimed to characterize student mask policies for US states and a sample of United States school districts at four points during the 2021–2022 school year and to determine whether urbanicity, demographics of district students, political affiliation of the state governor, or community viral transmission levels predicted the presence of a school mask mandate. We found that having a school mask mandate was related, most robustly, to the political affiliation of the state governor and urbanicity of the district, although these relationships changed over time.

Our findings echo prior literature noting the link between political affiliation and urbanicity to mask wearing, and the presence of statewide mitigation policies (10, 12, 13). However, this study extends prior findings to the school district setting where political affiliation and urbanicity similarly continued to influence mask policy at the school level.

While individual factors (namely, governor political affiliation, and urbanicity) were associated with the presence of district level student mask policy in September 2021, November 2021, and January 2022, there was no association between any of the factors we examined and the presence of a mask mandate in March 2022. By March 2022, all but 20 districts were either recommending mask wearing or making it optional. Of the remaining 20 districts that did not have an optional/recommended policy in March, the 12 that were in states with Democratic governors continued with their mask mandates. This could be due to lower COVID-19 case rates at this time, the February shift in CDC guidance, rising student vaccination coverage, or collective exhaustion with COVID-19 mitigation that shaped public and political appetites to continue masking requirements (24).

Generally, districts that served a larger proportion of students from minoritized racial/ethnic groups were more likely to have a mask mandate at any timepoint (upper third quartile of districts) and all (upper third and fourth quartiles of districts) timepoints. This is consistent with literature that describes high rates of mask wearing in communities of color overall (11) and could reflect greater demand for COVID-19 mitigation in communities disproportionately impacted by COVID-19 (7). Consistent with this hypothesis, Cowger et al. (4) found that schools in Massachusetts that sustained mask policies beyond when they were required tended to have greater proportions of Black and Latino students and staff.

We also found that, as the proportion of students with economic disadvantage increased, the odds of having a student mask mandate at all time points decreased. These results differ from Cowger et al. (4), who observed greater persistence of mask mandates in districts with a higher proportion of low-income students, but align with those of Kahane (10), who found that greater county-level median household income was associated with greater masking behavior. Our results should be interpreted in the context of a sample with relatively high average (64.8%) economic disadvantage based on our sampling strategy; further research should clarify the role of economic disadvantage in masking behavior and policy.

Notably, relative COVID-19 burden was not a significant predictor of a district mask mandate at each, any, or all of the four timepoints included in the analysis. This is salient as evidence indicates that school masking is an effective way to mitigate COVID-19 spread (25–27). However, what might be considered a directly relevant, public heath factor (COVID-19 prevalence) did not seem to be important in district mask policy decision making; district mask policies seem to be driven by factors such as political party, urbanicity, and local racial/ethnic composition, which in turn have tangible public health consequences for communities.

There are some limitations in our analysis. First, our school district level dataset was limited to data from 20 states, and purposive sampling was used within each state to select included districts. Relative county-level COVID-19 burden (high/medium/low by month) was used as a proxy for district COVID-19 incidence; however, in some places, school districts and counties are not interchangeable. In addition, our mask policy data are based on publicly available information from state and district websites and may not always reflect the most current policies that were being enforced.

Although mask wearing is an evidence-based intervention for reducing COVID-19 transmission, key questions remain about the degree to which school mask mandates impact the health of students, staff, and the community. Another important question is whether mandates negatively impact learning, socialization, or well-being and, if so, for which students. Our results raise ethical concerns about what factors should influence school health policies during a pandemic. Political affiliation of a state governor and district urbanicity may reflect community values about balancing protecting health with parental and student freedoms and other student interests, but they may also underplay or overplay the public health benefit, and the benefit to individual students and families, of mask mandates.

We found that various social, economic, and political factors predicted school mask policies for students, and that these associations varied over the course of the 2021–2022 school year. Our findings underscore the importance of learning from the experience of this pandemic to provide the best possible evidence of the benefits and harms of mask mandates. Although the next pandemic may have different epidemiologic and clinical features relevant to mask policy, a more robust evidence base will inform the development of more centralized, consistent guidance that may help narrow differences in mask policy by politics and geography. Additionally, public health officials should be mindful to deliver guidance in a way that anticipates and/or minimizes political reactions and enhances trust when attempting to influence school policy.

## Data availability statement

The original contributions presented in the study are included in the article/Supplementary material, further inquiries can be directed to the corresponding author.

## Author contributions

SJ, AA, KB, RF, MK, AN, AR, AT, and MC were instrumental to the creation of the dataset for analysis and the conception of the study. XG and LK led data analysis for the manuscript. LK led drafting and synthesis of results for the manuscript. All authors contributed to the article and approved the submitted version.

## Funding

LK, SJ, AA, KB, RF, XG, MK, AN, AR, AT, and MC disclosed receipt of the following financial support for the research, authorship, and/or publication of this article: Johns Hopkins eSchool+ Initiative.

## Conflict of interest

The authors declare that the research was conducted in the absence of any commercial or financial relationships that could be construed as a potential conflict of interest.

## Publisher’s note

All claims expressed in this article are solely those of the authors and do not necessarily represent those of their affiliated organizations, or those of the publisher, the editors and the reviewers. Any product that may be evaluated in this article, or claim that may be made by its manufacturer, is not guaranteed or endorsed by the publisher.
