# Supplementary material for: Predictors of student mask mandate policies in United States school districts during the COVID-19 pandemic
Source: Front Public Health. 2023 Jul 31;11:1217638. doi: 10.3389/fpubh.2023.1217638 (PMC10423804; doi:10.3389/fpubh.2023.1217638)
Supplement: Supplementary file 1 [file Table_1.docx]

***Supplementary Material***

**Predictors of student mask mandate policies in US school districts during the COVID-19 pandemic**

# **1 Supplementary Tables**

**Supplementary Table 1.** Fully-adjusted multivariable associations between school district level student mask mandate policy at each of four timepoints.

|  | **September 2021** ^b^ | | **November 2021** ^c^ | | **January 2022** ^d^ | | | **March 2022**  ^e^ | | | |  |
| --- | --- | --- | --- | --- | --- | --- | --- | --- | --- | --- | --- | --- |
|  | AOR | 95% CI | AOR | 95% CI | AOR | 95% CI | AOR | | | | 95% CI |  |
| **Governor Affiliation** ^a^ |  |  |  |  |  |  |  | | | |  |  |
| *Republican* | Reference | -- | Reference | -- | Reference | -- | Reference | | | | -- |  |
| *Democrat* | **39.49** | **2.96, 526.05** | 6.30 | 0.93, 42.79 | **17.95** | **2.13, 150.76** | 3.12 | | | | 0.82, 11.89 |  |
| **Urbanicity** ^a^ |  |  |  |  |  |  |  | | | |  |  |
| *Rural* | Reference | -- | Reference | -- | Reference | -- | Reference | | | | -- |  |
| *Non-rural* | 14.13 | 0.93, 213.64 | **36.49** | **1.93, 689.47** | **36.74** | **1.88, 718.15** | 2.27 | | | | 0.27, 18.91 |  |
| **Students from minoritized race/ethnic grp %** ^a^ |  |  |  |  |  |  |  | | | |  |  |
| *1^st^ quartile (7.1% to 26.0%)* | Reference | -- | Reference | -- | Reference | -- | Reference | | | | -- |  |
| *2^nd^ quartile (26.5% to 55.6%)* | 1.64 | 0.09, 31.02 | 1.30 | 0.10, 16.58 | 0.14 | 0.01, 2.50 | 0.28 | | | | 0.03, 2.70 |  |
| *3^rd^ quartile (61.3% to 85.2%)* | 11.95 | 0.29, 485.18 | 17.97 | 0.56, 578.17 | 5.56 | 0.15, 210.97 | 5.36 | | | | 0.21, 133.89 |  |
| *4^th^ quartile (86.8% to 99.9%)* | 22.64 | 0.26, 1966.56 | 16.21 | 0.25, 1058.72 | 2.77 | 0.04, 198.57 | 10.53 | | | | 0.27, 413.14 |  |
| **Students with economic disadvantage %** | 0.99 | 0.94, 1.04 | 0.98 | 0.93, 1.03 | 0.98 | 0.94, 1.04 | 0.97 | | | | 0.93, 1.02 |  |
| **County-level COVID-19 Incidence** ^a^ |  |  |  |  |  |  |  | | | |  |  |
| *Low* | Reference | -- | Reference | -- | Reference | -- | Reference | | | | -- |  |
| *Medium* | 3.53 | 0.35, 35.07 | 2.90 | 0.38, 22.38 | 0.47 | 0.05, 4.48 | 0.26 | | | | 0.05, 1.33 |  |
| *High* | 0.74 | 0.06, 8.57 | 9.24 | 0.66, 130.07 | 0.71 | 0.04, 11.26 | 1.01 | | | | 0.19, 5.29 |  |
|  |  |  |  |  |  |  |  | |  |  | | |

AOR: adjusted odds ratio; CI: confidence interval

^a^ Bold font indicates statistically significant associations at P<.05 level.

^b^ Multivariable regression model pseudo-R^2^=0.5046.

^c^ Multivariable regression model pseudo-R^2^=0.4237.

^d^ Multivariable regression model pseudo-R^2^=0.4355.

^e^ Multivariable regression model pseudo-R^2^=0.1998.

**Supplementary Table 2.** Univariate associations and fully-adjusted multivariable associations between school district teacher mask mandate policy at any one time point (September 2021, November 2021, January 2022, and March 2022).

|  | Teacher Mask Mandate | | | |
| --- | --- | --- | --- | --- |
|  | Univariate | | Multivariable ^b^ | |
|  | OR | 95% CI | AOR | 95% CI |
| Governor Affiliation ^a^ |  |  |  |  |
| *Republican* | Reference | -- | Reference | -- |
| *Democrat* | **3.82** | **1.64, 8.91** | **3.71** | **1.51, 9.12** |
| Urbanicity ^a^ |  |  |  |  |
| *Rural* | Reference | -- | Reference | -- |
| *Non-rural* | **6.04** | **1.85, 19.66** | **4.63** | **1.24, 17.33** |
| Students from minoritized race/ethnic grp % ^a^ |  |  |  |  |
| *1^st^ quartile (7.1% to 26.0%)* | Reference | -- | Reference | -- |
| *2^nd^ quartile (26.5% to 55.6%)* | 1.43 | 0.43, 4.72 | 1.26 | 0.29, 5.46 |
| *3^rd^ quartile (61.3% to 85.2%)* | 3.22 | 0.97, 10.74 | **5.98** | **1.28, 28.02** |
| *4^th^ quartile (86.8% to 99.9%)* | 3.55 | 1.05, 12.00 | **9.01** | **1.38, 58.93** |
| Students with economic disadvantage % | 1.00 | 0.99, 1.02 | 0.98 | 0.96, 1.01 |
| County-level COVID-19 Incidence |  |  |  |  |
| *Low* | Reference | -- | Reference | -- |
| *Medium* | 1.18 | 0.62, 2.24 | 1.27 | 0.55, 2.94 |
| *High* | 1.56 | 0.93, 2.64 | 1.80 | 0.90, 3.62 |

OR: odds ratio; CI: confidence interval; AOR: adjusted odds ratio; QIC: quasi likelihood under the independence model criterion

^a^ Bold font indicates statistically significant associations at P<.05 level.

^b^ Multivariable generalized estimating equation model QIC=256.105.

**Supplementary Table 3.** Univariate associations and fully-adjusted multivariable associations between school district level teacher mandate policy at all four time points of September 2021, November 2021, January 2022, and March 2022.

|  | Teacher Mask Mandate | | | |
| --- | --- | --- | --- | --- |
|  | Univariate | | Multivariable ^b^ | |
|  | OR | 95% CI | AOR | 95% CI |
| Governor Affiliation ^a^ |  |  |  |  |
| *Republican* | Reference | -- | Reference | -- |
| *Democrat* | **3.43** | **1.90, 6.19** | **3.56** | **1.89, 6.70** |
| Urbanicity ^a^ |  |  |  |  |
| *Rural* | Reference | -- | Reference | -- |
| *Non-rural* | **2.86** | **1.26, 6.48** | 2.07 | 0.80, 5.35 |
| Students from minoritized race/ethnic grp % ^a^ |  |  |  |  |
| *1^st^ quartile (7.1% to 26.0%)* | Reference | -- | Reference | -- |
| *2^nd^ quartile (26.5% to 55.6%)* | 1.00 | 0.41, 2.47 | 0.87 | 0.30, 2.47 |
| *3^rd^ quartile (61.3% to 85.2%)* | **2.75** | **1.20, 6.30** | **4.37** | **1.01, 18.89** |
| *4^th^ quartile (86.8% to 99.9%)* | **2.75** | **1.20, 6.30** | **5.73** | **1.06, 30.83** |
| Students with economic disadvantage % | 1.01 | 1.00, 1.02 | 0.99 | 0.97, 1.01 |
| County-level COVID-19 Incidence |  |  |  |  |
| *Low* | Reference | -- | Reference | -- |
| *Medium* | 0.69 | 0.35, 1.35 | 0.78 | 0.37, 1.65 |
| *High* | 0.77 | 0.39, 1.53 | 0.81 | 0.38, 1.72 |

OR: odds ratio; CI: confidence interval; AOR: adjusted odds ratio

^a^ Bold font indicates statistically significant associations at P<.05 level.

^b^ Multivariable regression model pseudo-R^2^=0.1033.
